# Batteryless IoT Sensing Using Thermoelectric Energy Harvesting from Industrial Motor Waste Heat

**DOI:** 10.3390/s26051644

**Published:** 2026-03-05

**Authors:** Kamil Bancik, Jaromir Konecny, Martin Stankus, Radim Hercik, Jiri Koziorek, Vytautas Markevičius, Darius Andriukaitis, Michal Prauzek

**Affiliations:** 1 Department of Cybernetics and Biomedical Engineering, VSB—Technical University of Ostrava, 17. listopadu 2172/15, 708 00 Ostrava-Poruba, Czech Republic; kamil.bancik@vsb.cz (K.B.); jaromir.konecny@vsb.cz (J.K.); martin.stankus@vsb.cz (M.S.); radim.hercik@vsb.cz (R.H.); jiri.koziorek@vsb.cz (J.K.); 2 Department of Electronics Engineering, Faculty of Electrical and Electronics Engineering, Kaunas University of Technology, K. Donelaicio g. 73, 44249 Kaunas, Lithuania; vytautas.markevicius@ktu.lt (V.M.); darius.andriukaitis@ktu.lt (D.A.)

**Keywords:** thermoelectric generator, energy harvesting, waste heat recovery, battery-free sensing, predictive maintenance, Industry 4.0

## Abstract

This study presents the design, implementation, and validation of a thermoelectric energy harvesting system that exploits waste heat from an industrial electric motor to power an autonomous wireless sensor device. The proposed prototype integrates a single thermoelectric generator directly onto the motor housing and leverages the built-in cooling fan to maintain a stable thermal gradient of approximately 4–5 °C. Under real factory conditions, the system harvested 6.17 J of energy over 9612 s, sustaining continuous operation and 41 successful Long Range (LoRa) data transmissions with a positive energy balance. Compared with related works, the prototype achieved competitive or superior performance while operating at a lower motor rating of 0.25 kW, highlighting its efficiency relative to system scale. Key innovations include a hybrid DC/DC conversion chain bridging ultra-low input voltages to modern microcontrollers, and an adaptive transmission strategy that ensures predictable energy management and reliable wireless communication. These results demonstrate the feasibility of battery-free sensing in industrial environments and underline the potential of thermoelectric harvesting as a cost-effective, maintenance-free, and environmentally responsible solution for predictive maintenance and Industry 4.0 applications.

## 1. Introduction

In modern industrial systems, the demand for continuous data acquisition has become a fundamental requirement for ensuring operational efficiency, safety, and competitiveness [1,2]. The transition towards digitalized production, often framed under the concepts of Industry 4.0 and the Internet of Things (IoT), increasingly relies on advanced sensing technologies that enable real-time monitoring of machines and processes [3,4]. Artificial intelligence methods play a pivotal role in this transformation, providing the means for predictive maintenance and condition monitoring that reduce unplanned downtime, extend the service life of critical components, and optimize resource utilization [5]. At the same time, ecological and economic pressures are driving the development of sustainable solutions that minimize dependence on disposable batteries and promote energy-efficient maintenance practices, thereby lowering both operational costs and environmental impact [6].

Despite the rapid progress in sensing and communication technologies, the long-term deployment of IoT devices remains constrained by their energy supply [7]. Conventional solutions rely on primary or rechargeable batteries, which introduce several limitations: restricted lifetime, the need for periodic replacement or recharging, and the associated maintenance costs. In many industrial applications, the encapsulation of sensing devices makes battery access impractical or even impossible. Moreover, the large-scale use of batteries contributes to ecological concerns related to material consumption and waste management. To address these challenges, research has increasingly focused on energy harvesting strategies, which exploit ambient energy sources to achieve autonomous operation of wireless sensor systems [8].

Among the available energy harvesting approaches, thermal energy has attracted significant attention due to its ubiquity in both natural and industrial environments. Heat sources such as solar radiation, geothermal reservoirs, or waste heat from machinery represent promising opportunities for generating electrical power in situ [1,9]. In particular, waste heat from electric motors and other industrial equipment offers a stable and predictable energy source, making it highly suitable for long-term sensor operation. Thermoelectric generators (TEGs) provide a robust means of exploiting such temperature gradients, as they operate without moving parts, require minimal maintenance, and can deliver continuous power even from small temperature differences [10]. These properties make TEG-based harvesters especially attractive for industrial IoT applications where reliability and autonomy are critical.

**Figure 1:**
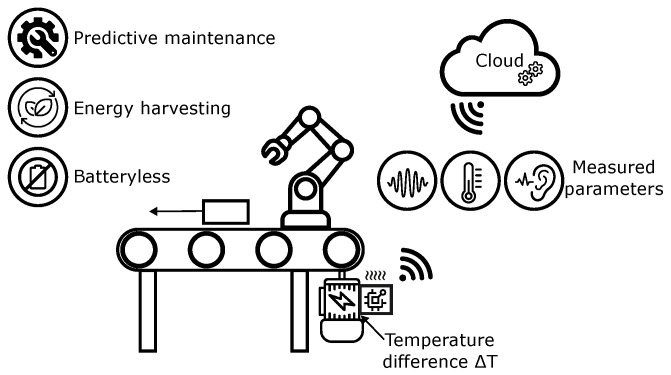
Application concept of a thermoelectric energy-harvesting IoT sensor in a smart factory environment. Waste heat from an electric motor is converted into electrical energy that powers a wireless node. The collected data are transmitted to the cloud, enabling artificial intelligence methods for predictive maintenance and condition monitoring.

The proposed concept builds on these principles by integrating a TEG directly with an industrial electric motor, thereby harvesting waste heat to supply energy for an autonomous IoT node. As illustrated in Figure 1, the harvested energy powers a wireless sensor system capable of measuring operational parameters of the motor and transmitting the data to the cloud. This architecture eliminates the dependence on batteries, ensuring sustainable and maintenance-free operation. By enabling continuous monitoring of machinery, the solution supports both predictive maintenance and condition monitoring, which are key pillars of modern smart manufacturing strategies. The integration with cloud-based analytics further allows advanced data processing, including the application of artificial intelligence methods for fault detection and performance optimization.

This study advances the field of energy-autonomous sensing by demonstrating the practical integration of thermoelectric harvesting with industrial equipment. Unlike previous works that primarily focus on laboratory characterization of TEG modules or partial subsystems, the presented solution delivers a fully functional prototype of an IoT device powered exclusively by harvested thermal energy. The system shows that reliable wireless communication and cloud connectivity can be achieved without batteries, supporting long-term predictive maintenance and condition monitoring in real industrial environments.

The main contributions of this work are as follows:

1. Develop a thermoelectric energy harvesting approach that exploits waste heat from industrial motors through optimized mechanical integration.
2. Implement a complete prototype that directly powers an IoT device from harvested energy without the use of batteries.
3. Demonstrate reliable wireless sensing with cloud data transmission supporting both predictive maintenance and condition monitoring in real industrial conditions.

The remainder of this paper is structured as follows: Section 2 describes the design of the proposed thermoelectric energy harvesting system and its integration with the motor. Section 3 presents the experimental methodology, including the setup and measurement procedures. Section 4 summarizes the results obtained from both the harvester evaluation and the operation of the sensor device. Section 5 provides a detailed discussion, comparing the findings with related work, outlining the main contributions, limitations, and future research directions. Finally, Section 6 concludes the paper by highlighting the key outcomes and practical implications of the study.

For clarity and consistent notation, the main thermal and electrical parameters used throughout the manuscript are summarized in Nomenclature.

## 2. Related Work

In the context of Industry 4.0, continuous data acquisition from machines and processes is essential for effective production planning and maintenance [1,2]. Wired sensor systems have traditionally been used for this purpose, since they provide both power and data transmission. Their disadvantage is limited flexibility, especially in distributed or hard-to-reach locations. Wireless sensor nodes address this limitation and enable easier deployment [3,4], but they usually rely on batteries that require regular replacement or recharging. In many cases, access to batteries is difficult or impractical, and large numbers of deployed batteries also raise environmental concerns. To overcome these limitations, research has focused on energy harvesting, where ambient energy is converted into electrical power for long-term autonomous sensor operation [8].

A wide range of studies has investigated thermoelectric energy harvesting for powering wireless sensor nodes in different environments and applications. Table 1 summarizes representative experiments, including gearbox monitoring [11], railway bearings [12], vehicle exhaust systems [13], and electric motors in industrial settings [14,15,16,17]. Other works have targeted power generation from heating elements in vehicles [18], switch cabinets [19], coking plants [20], thermos pots [21], and soil–air gradients for outdoor IoT devices [22,23]. Additional applications include respiration monitoring using micro-TEGs [24], hybrid TEG/phase change materials (TEG/PCM) systems [25], flexible generators powered by body heat [26], and buried infrastructure monitoring with long-term underground deployments [27]. The reported results vary widely in terms of available temperature differences, harvested energy, and achieved output power, reflecting both the diversity of operating conditions and the limitations of current solutions.

As seen in Table 1, the reported temperature differences range from only a few degrees Celsius in small motors or soil–air systems [14,16,22] up to several tens of degrees in large engines or industrial processes [13,20,25]. The corresponding harvested power varies from hundreds of microwatts to several watts. Many studies confirm that even small ΔT values are sufficient to enable low-power sensing, but most experiments remain limited to laboratory settings or partial demonstrations of energy conversion. Only a few works [14,15,16,17] attempt to integrate TEG harvesters with complete IoT sensor nodes, and these typically provide restricted functionality or short-term operation. This highlights the challenge of achieving reliable, batteryless sensing under real industrial conditions.

Iezzi [28] evaluates a TEG mounted on pipe insulation, where only a very small portion of the heat is available for conversion, resulting in sub-milliwatt power levels. Jiang [29] does not analyze an operational system but rather characterizes the behavior of the TEG itself under generic heating, providing electrical measurements without integrating the harvester into an application. In contrast, Aragonés [30] and Boegel [31] exploit high-grade thermal sources from steam pipelines or coolant lines, where temperature gradients of tens or even hundreds of degrees Celsius enable orders-of-magnitude higher output, reaching the watt range in controlled setups. The most practically relevant example for low-grade industrial waste heat is Santos [14], who demonstrate that even a modest temperature difference of 3.27 °C on an electric motor can yield 3.19 mW (TEG 55 × 55 mm) and accumulate 0.909 J every five min sufficient to power a complete wireless vibration-monitoring node.

In summary, previous research demonstrates the feasibility of thermoelectric harvesting for supplying low-power electronics, but most studies are restricted to laboratory validation, simplified setups, or short-term demonstrations. Only a limited number of works combine TEGs with wireless nodes in real operating environments, and these often face constraints in harvested power, stability, or long-term applicability. There remains a clear need for practical evaluation of complete IoT devices powered directly from industrial waste heat, with robust data transmission and integration into predictive maintenance and condition monitoring frameworks. The present study addresses this gap by implementing and validating a fully functional prototype in a smart factory environment.

From a long-term deployment perspective, industrial motors are also a source of continuous mechanical vibration, which may affect the stability of the motor/HTP/TEG interfaces over time (e.g., gradual loosening, micro-slip, or “interface walk-out”), potentially increasing the effective contact thermal resistance and reducing the achievable ΔT. In the presented proof-of-concept, the validation window was limited and a dedicated endurance campaign (multi-day vibration exposure, thermal cycling, and aging of the interface materials) was not performed. Nevertheless, the mechanical concept is intended to maintain consistent thermal contact by using a curvature-matched rigid adapter and a clamped assembly that provides sustained normal force, while thermal paste is used to fill microscopic voids and reduce sensitivity to surface roughness. Compared to purely adhesive attachment, a mechanically clamped interface is less sensitive to viscoelastic creep and can be periodically re-torqued during maintenance if required. In future work, we will quantify long-term stability by extended operation tests including vibration and thermal cycling, and we will evaluate alternative compliant interlayers (e.g., pads/graphite sheets) that may further improve vibration tolerance while preserving low contact resistance.

## 3. Methods and Experiment

This section outlines the methodology applied to design, implement, and validate the proposed thermoelectric energy-harvesting system integrated with an industrial motor. The presentation follows a structured approach: first, the construction of the waste heat energy harvester is introduced, including mechanical design considerations and integration with the motor housing. Second, the development of a batteryless IoT prototype is described, highlighting the energy conversion chain and wireless communication module. Finally, the experimental procedure is detailed, covering the Smart Factory test environment, measurement chain, and test scenarios for both electrical characterization and real IoT operation. Together, these subsections provide a comprehensive account of the system development and its validation under realistic industrial conditions.

### 3.1. Waste Heat Energy Harvester for Electrical Motor

To obtain a usable temperature difference for thermoelectric energy harvesting, a prototype was designed for integration with a small industrial motor operating in the Smart Factory demonstration line. The selected unit is a BOSCH (Germany) Rexroth type 3 842 547 992 motor (0.25 kW, worm gear) driving one of the conveyor systems. Since the thermal field on the motor housing is not uniform, an infrared analysis was carried out to locate the most suitable area for harvester installation.

**Figure 2:**
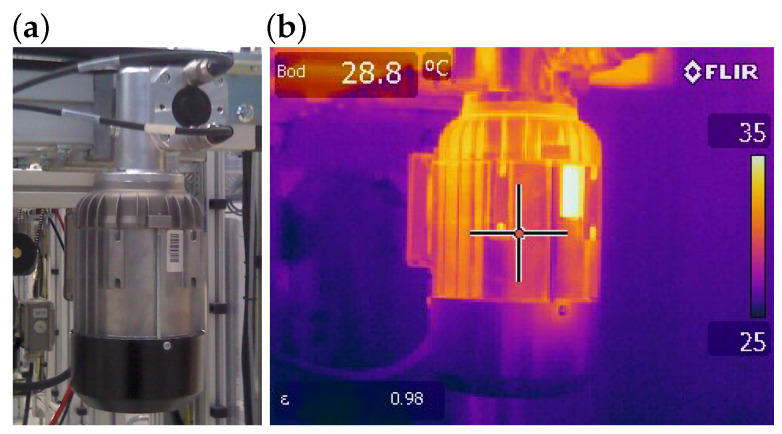
An electric motor captured by a conventional camera and its corresponding infrared image obtained using a thermal camera, illustrating the maximum measured surface temperature: (**a**) photograph and (**b**) infrared thermographic image.

Figure 2 shows the surface temperature distribution, highlighting hot spots on the smooth part of the housing where the thermoelectric module can be mounted to achieve a stable temperature gradient during operation. In practice, the measured surface temperatures ranged from approximately 35 °C to 60 °C, providing a sufficient gradient against the ambient air to enable effective energy conversion.

**Figure 3:**
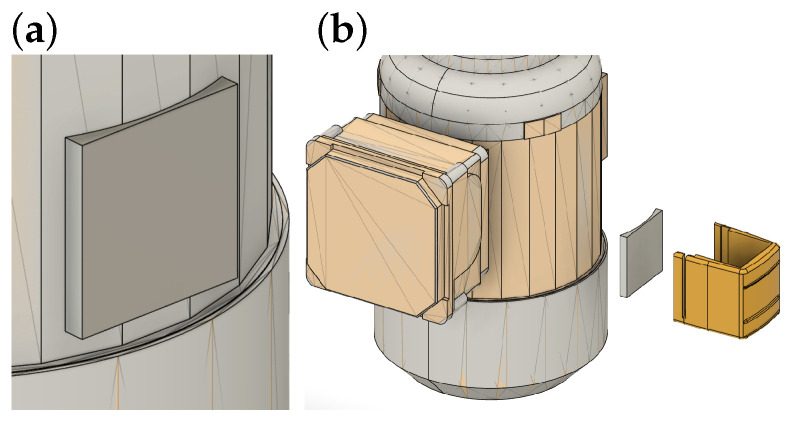
Visualization of HTP arrangement surrounding the motor, including the configuration of HTP components and the air tunnel: (**a**) detail view and (**b**) exploded view of the assembly.

The mechanical construction of the harvester was designed to enable reliable attachment of a flat TEG onto the curved surface of the motor casing. To compensate for the curvature, a custom-machined aluminum adapter, further referred to as the HTP, was introduced as an intermediate element ensuring uniform contact and efficient heat transfer. The selected TEG was a Marlow (Dallas, TX, USA) RC12-8-01LS module (40 mm × 44.7 mm, thickness 3.51 mm), chosen based on its proven performance in previous experiments. On the cold side, a Wakefield Thermal (Nashua, NH, USA) 910-40-2-33-2-B-0 aluminum heatsink was mounted, matching the footprint of the TEG. The heatsink was equipped with tubular fins to enlarge the effective surface area and reduce thermal resistance. To minimize contact imperfections at each interface, all surfaces between the motor, HTP, TEG, and heatsink were coated with ARCTIC (Brunswick, Germany) MX-4 thermal paste. While thermal paste improves initial contact quality, its properties can degrade over long-term operation due to aging mechanisms such as drying, pump-out under vibration, and micro-cracking induced by thermal cycling. Because the available temperature difference is only a few degrees Celsius, the thermal contact resistance at the motor/HTP and HTP/TEG interfaces can become a critical factor. In this proof-of-concept prototype, we did not perform a dedicated separation of individual interface temperature drops; however, an order-of-magnitude upper bound can be estimated for the interfacial layer. Assuming the thermal paste forms a thin layer of thickness *t* on a contact area *A*, its thermal resistance can be approximated as

(1) $$R_{\mathrm{paste}}\approx \frac{t}{kA},$$

where *k* is the effective thermal conductivity of the paste. For the used module footprint ($A\approx 40\;\mathrm{mm}\times 44.7\;\mathrm{mm}\approx 1.79\times 10^{-3}\;m^{2}$) and a conservative layer thickness of t = 0.1–0.2 mm, $R_{\mathrm{paste}}$ remains on the order of 0.01–0.04 K/W for typical paste conductivities. Even for an unrealistically high heat flow of 10 W through the interface, this corresponds to a temperature drop below approximately 0.1–0.4 K per interface, i.e., markedly smaller than the measured 4–5 °C gradient across the TEG during operation. In practice, the dominant uncertainty is the microscopic metal-to-metal contact (surface roughness and residual voids), which is mitigated by (i) a custom-machined adapter matching the motor curvature to maximize real contact area, (ii) controlled clamping of the assembly, and (iii) thermal paste filling microgaps. We therefore treat the presented results as a system-level validation in which the measured ΔT already implicitly reflects the combined effect of all interfaces. A dedicated interface characterization using additional temperature taps across the motor/HTP and HTP/TEG interfaces will be addressed in future work. The arrangement of the HTP components and the air tunnel is illustrated in Figure 3.

In the low-ΔT regime, a part of the heat can bypass the thermoelectric legs through unintended parallel paths (e.g., mounting components and stagnant air volumes around the module), which effectively reduces the temperature drop experienced by the active material. To quantify this effect, we represent the intended heat flow path through the packaged TEG as a thermal resistance $R_{\mathrm{TEG}}$ in parallel with an aggregate parasitic resistance $R_{\mathrm{par}}$ capturing the bypass paths. Under steady-state conditions,

(2) $${\dot{Q}}_{\mathrm{TEG}}=\frac{\Delta T_{\mathrm{env}}}{R_{\mathrm{TEG}}},\;{\dot{Q}}_{\mathrm{par}}=\frac{\Delta T_{\mathrm{env}}}{R_{\mathrm{par}}},$$

which yields the fraction of heat passing through the thermoelectric path

(3) $$\eta_{\mathrm{TEG}}≜\frac{{\dot{Q}}_{\mathrm{TEG}}}{{\dot{Q}}_{\mathrm{TEG}}+{\dot{Q}}_{\mathrm{par}}}=\frac{R_{\mathrm{par}}}{R_{\mathrm{TEG}}+R_{\mathrm{par}}}.$$

Thermal shunting is suppressed when $R_{\mathrm{par}}\gg R_{\mathrm{TEG}}$. A conservative upper bound for the dominant parasitic contribution can be obtained by considering stagnant air bypass conduction: for an air gap of thickness *g* conducting across an effective bypass area $A_{\mathrm{gap}}$, $R_{\mathrm{air}}\approx g/\left(k_{\mathrm{air}}A_{\mathrm{gap}}\right)$. Even for pessimistic millimeter-scale gaps and centimeter-scale bypass areas, $R_{\mathrm{air}}$ is typically much higher than the effective resistance of a packaged TEG module of comparable footprint, implying that $\eta_{\mathrm{TEG}}$ remains close to unity (e.g., $R_{\mathrm{par}}/R_{\mathrm{TEG}}=10$ already gives $\eta_{\mathrm{TEG}}\approx 0.91$).

In addition to the inherently high resistance of stagnant air, the prototype layout was designed to minimize conductive shunts: (i) the hot-side adapter is machined to match the motor curvature, reducing uncontrolled air volumes, (ii) controlled clamping and thermal paste minimize microgaps at interfaces, (iii) hot-side and cold-side metallic parts are geometrically separated so that the primary conductive path between them is through the TEG footprint, and (iv) the fan-assisted air tunnel increases cold-side convection, helping to maintain the effective gradient across the module.

**Figure 4:**
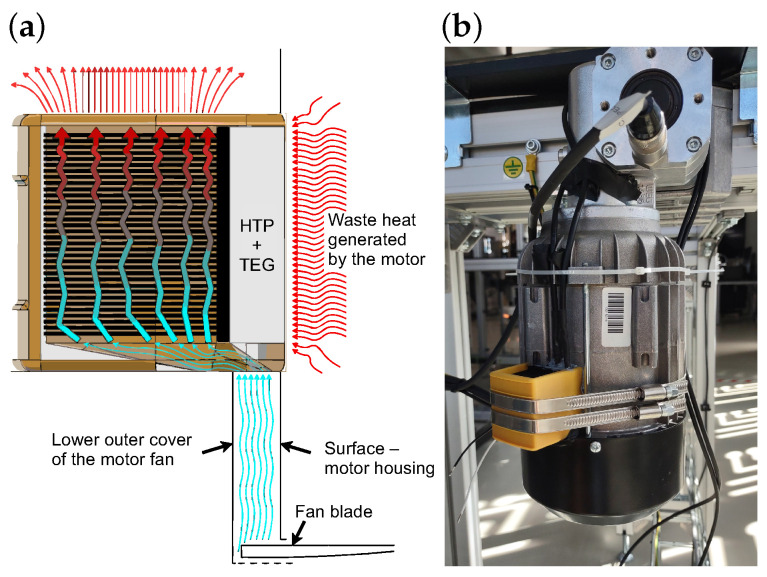
(**a**) Proposed air tunnel design illustrating airflow, waste heat transfer, and the integration of HTP and TEG modules with the motor structure. (**b**) Deployment of the energy harvesting device on the small electrical motor in the Industry 4.0 demonstrator line, equipped with temperature sensors for real-world validation.

To further enhance the thermal gradient across the TEG, the design also takes advantage of the airflow generated by the integrated cooling fan of the motor. During operation, the fan drives air through the housing, which is partially finned to improve heat dissipation. The harvester was mounted on a smooth section of the housing without fins, where localized heat accumulates. This position simultaneously enables the directed airflow to pass through the added air tunnel surrounding the heatsink, as illustrated in Figure 4. The accelerated cooling of the heatsink lowers its surface temperature and thus increases the ΔT across the TEG. Figure 4b shows the deployment of the complete energy harvesting device on the motor in the Industry 4.0 demonstrator line, where it was also equipped with temperature sensors to enable detailed validation under real operating conditions.

### 3.2. Prototype of Batteryless IoT Device

**Figure 5:**
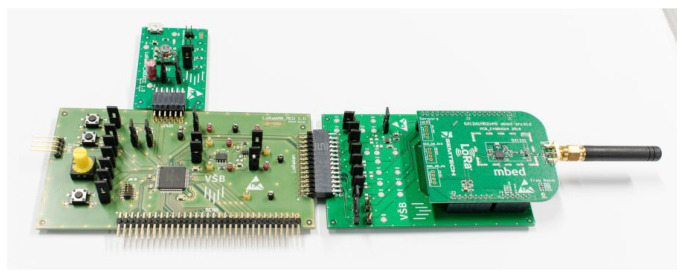
Prototype of the IoT node powered by thermoelectric energy harvesting. The hardware integrates: LTC3109 (Linear Technologies, Milpitas, CA, USA) DC/DC converter with 20 mF storage capacitor, LMR1901YG-M (ROMH Co., Ltd., Kyoto, Japan) operational amplifier, KL25Z4 (NXP Semiconductor, Eindhoven, The Netherlands) microcontroller, TPS62840 (Texas Instruments, Dallas, TX, USA) step-down converter, and SX1261 (Semtech Corp., Camarillo, CA, USA) LoRa transceiver.

A fully operational prototype of a wireless IoT node was developed to demonstrate the feasibility of powering embedded electronics exclusively from thermoelectric energy harvesting (Figure 5). The design objective was to achieve reliable operation with minimal energy overhead while maintaining compatibility with industrial deployment scenarios.

The harvested voltage from the TEG is conditioned by an LTC3109 (Linear Technologies, Milpitas, CA, USA) low-voltage DC/DC converter, which charges a 20 mF storage capacitor to buffer the intermittent energy supply. An LMR1901YG-M (ROMH Co., Ltd., Kyoto, Japan) operational amplifier is included to enable precise voltage measurement and monitoring of the storage element. Downstream regulation is provided by a TPS62840 (Texas Instruments, Dallas, TX, USA) high-efficiency buck converter, delivering a stable supply to the digital electronics. This configuration ensures reliable bridging between the millivolt-level TEG output and the requirements of modern low-power components.

The control and sensing logic are executed by an NXP (NXP Semiconductor, Eindhoven, The Netherlands) KL25Z4 microcontroller, a 32-bit ARM Cortex-M0+ device with integrated peripherals for data acquisition and energy management. For long-range wireless communication, a Semtech (Camarillo, CA, USA) SX1261 Long Range (LoRa) transceiver is employed, enabling periodic transmission of measured data to the cloud. LoRa technology was selected for its favorable balance of range and energy efficiency, making it well suited for batteryless IoT devices powered by energy harvesting. The modular design further allows adaptation of transmission parameters (e.g., spreading factor, bandwidth, payload size) to match the available harvested energy, as demonstrated in [32].

To ensure reproducibility, the LoRa radio configuration used in the experiments is reported explicitly. The SX1261 transceiver was operated in the EU868 band with the transmit power set to the maximum EIRP permitted by the EU868 regional configuration (as enforced by the LoRaWAN stack). For all reported experiments, the LoRa modulation parameters were kept constant at SF12 and BW = 125 kHz. The preamble length was set to 8 symbols and the payload size to 32 bytes.

### 3.3. Experimental Procedure

The experimental campaign was designed with two main objectives: first, to characterize the thermal and electrical behavior of the proposed harvester under real operating conditions of an industrial motor; and second, to verify its capability to reliably power a wireless IoT node for long-term autonomous operation. For this purpose, a dedicated measurement setup was established within the Smart Factory demonstrator line at VSB–Technical University of Ostrava. The environment provides realistic conditions for testing energy harvesting from industrial motors, while allowing controlled monitoring of thermal fields, harvested power, and wireless data transmission performance.

**Figure 6:**
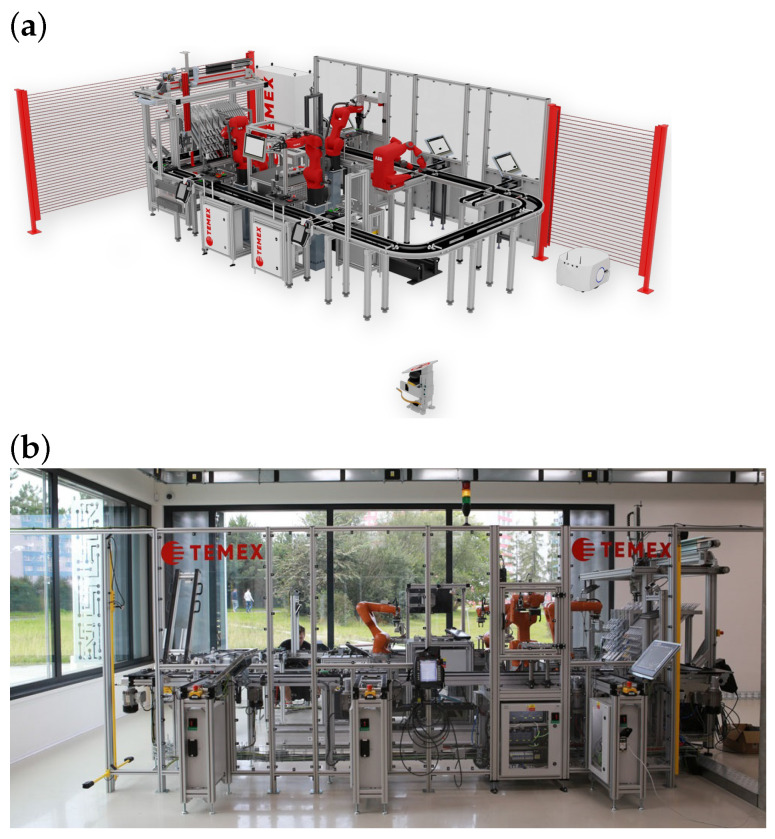
Experimental test bed of a Smart Factory established at VSB–Technical University of Ostrava, providing the environment for energy harvesting experiments: (**a**) 3D model, (**b**) photograph.

The Smart Factory demonstrator (Figure 6) consists of a modular production line equipped with conveyor systems, robotic manipulators, and multiple electric drives. This platform is primarily used for education and research in the field of Industry 4.0, providing a realistic environment for testing advanced sensing and energy harvesting concepts. The harvester prototype was installed on a BOSCH Rexroth 0.25 kW motor driving one of the conveyor modules. This integration allowed continuous exposure to realistic thermal loads generated during operation, while also enabling straightforward instrumentation for experimental measurements.

**Figure 7:**
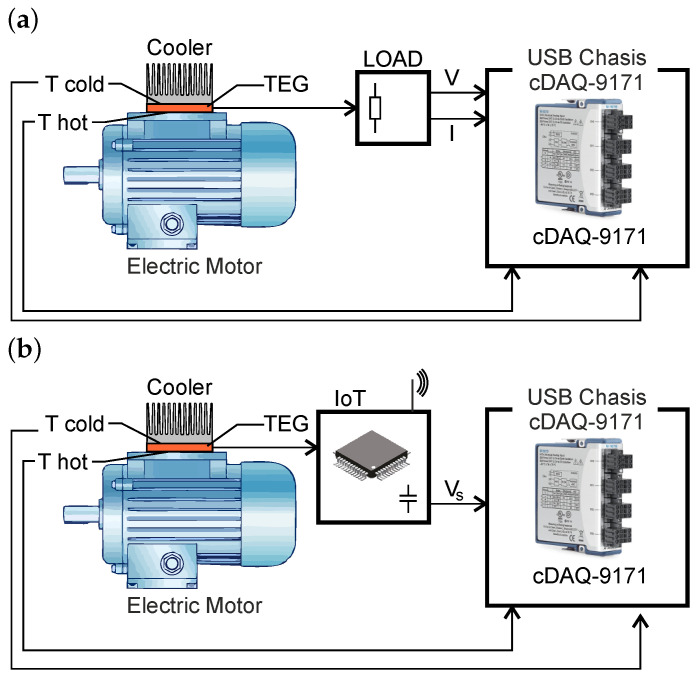
Experimental setup using motor waste heat to supply a TEG: (**a**) characterization with a resistive load and (**b**) operation with an IoT node powered via a storage capacitor for wireless transmission.

To capture the operating characteristics of the TEG, a dedicated measurement chain was implemented. Surface temperatures on the hot and cold sides of the TEG were monitored using Pt100 sensors (type TT-PT100A-2050-11-AUNI (Tewa Temperature Sensors Ltd., Lublin, Poland), class A accuracy) connected to a National Instruments (NI) (Austin, TX, USA) NI-9219 universal DAQ module via a four-wire interface to ensure high precision. The DAQ module, housed in a NI cDAQ-9171 chassis, provided both power and data communication with a host PC, while a custom LabVIEW application was developed for data acquisition, processing, and visualization. Two complementary test configurations were applied (Figure 7): (a) measurements with a resistive load matched to the TEG internal resistance, and (b) direct powering of the IoT node through the storage capacitor to verify wireless transmission under realistic operating conditions.

To analyze the electrical behavior of the system in greater detail, two circuit configurations were implemented. The first configuration (Figure 7a) connected the TEG output to an LTC3109 low-voltage DC/DC converter, which ensured efficient energy transfer and enabled operation from temperature differences as low as ±1 °C. This setup allowed precise evaluation of the conversion efficiency and the behavior of the storage capacitor during charging. The second configuration (Figure 7b) included the complete IoT node, where the harvested energy powered the KL25Z4 microcontroller and the SX1261 LoRa transceiver. In this case, the LTC3109 supplied the baseline energy management, while the TPS62840 high-efficiency step-down converter provided stable power to the radio during transmission. This dual approach enabled both laboratory-style characterization and verification of real IoT operation under energy harvesting conditions.

The IoT node operated under a simple duty-cycling strategy implemented in the microcontroller firmware. The algorithm continuously monitored the capacitor voltage and initiated a wireless transmission only when the voltage exceeded 2.0 V, ensuring that sufficient energy was available to complete the operation. After each successful transmission, the node entered a sleep mode for 20 s to minimize quiescent consumption and allow the capacitor to recharge from the harvester. This strategy guaranteed reliable operation of the wireless link while adapting the duty cycle to the variable amount of harvested energy.

## 4. Results

The experimental evaluation was carried out in two consecutive stages to verify both the performance of the developed thermoelectric energy harvesting device and its capability to reliably power an IoT sensor node. First, the energy harvester prototype was analyzed in terms of thermal and electrical behavior under realistic operating conditions. Subsequently, the feasibility of autonomous operation of a wireless sensor node powered solely by harvested energy was assessed, focusing on transmission regularity, energy balance, and system stability.

### 4.1. Energy Harvester Performance

To assess the capability of the developed thermoelectric prototype to convert waste heat into usable electrical energy, the device was mounted on a small electric motor driving a manipulator in the production line test bed. The objective of this evaluation was twofold: to characterize the thermal behavior of the harvester under realistic operating conditions and to quantify the corresponding electrical output as a function of the temperature difference across TEG.

**Figure 8:**
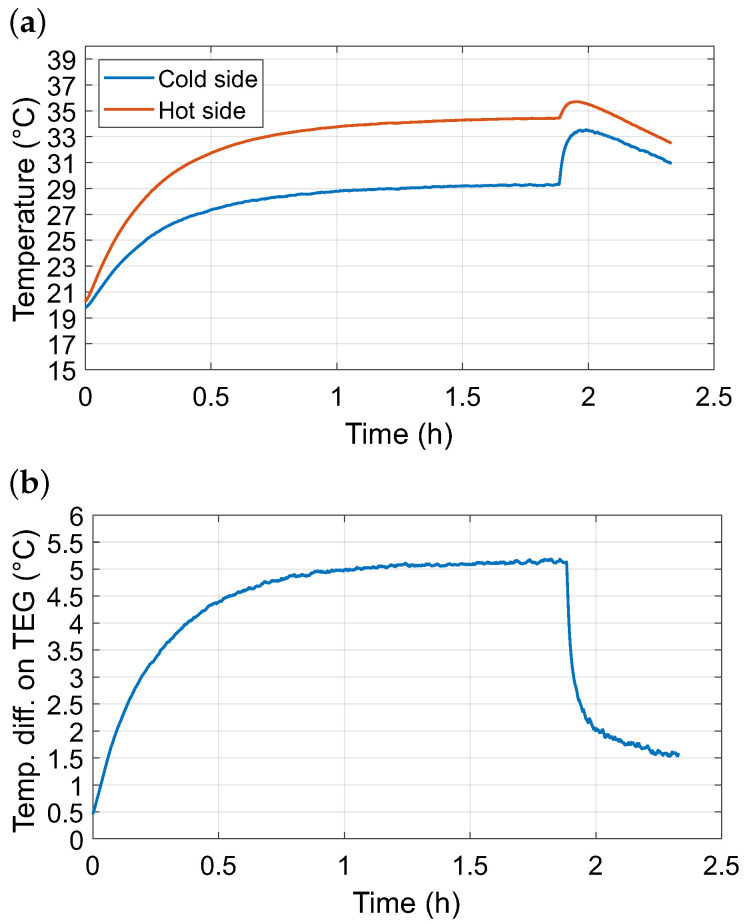
(**a**) Temperatures recorded during the experiment on the hot and cold sides of the TEG placed in a energy harvesting device. (**b**) Temperature difference on TEG during the experiment with a small electrical motor.

Figure 8a depicts the temperature evolution on the hot and cold sides of the TEG during continuous motor operation. After start-up, both surfaces exhibited a gradual increase in temperature, reflecting the heating of the motor casing and the heat sink. The resulting temperature difference stabilized in the range of 4.8–5.2 °C, with an average of 4.0 °C over the entire experiment (Figure 8b). A transient increase was observed immediately after the motor was switched off, caused by the cessation of the integrated cooling fan, which temporarily reduced convective heat dissipation before the system returned to ambient conditions. These findings demonstrate that a measurable and stable thermal gradient can be established even without dedicated thermal management.

**Figure 9:**
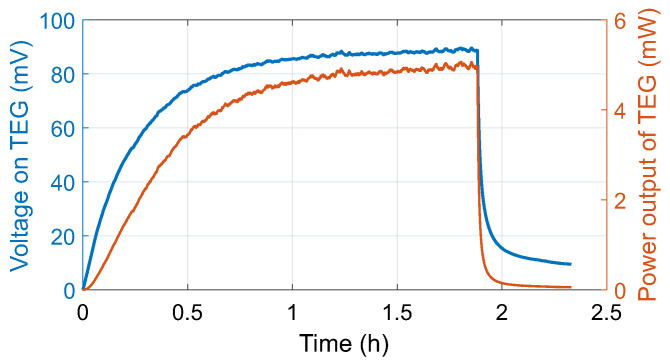
Measured voltage and calculated power during prototype validation.

The corresponding electrical characteristics are shown in Figure 9. The TEG produced a voltage that increased with time until thermal steady state was reached. The maximum measured voltage was 89.6 mV, with a mean of 64.2 mV and a median of 81.5 mV. The associated electrical power output peaked at 5.07 mW, with an average value of 3.16 mW and a median of 4.19 mW. The observed temporal evolution of both voltage and power closely followed the thermal response of the system, indicating that the harvester output becomes progressively more stable as the thermal conditions approach equilibrium.

A more detailed view of the harvester behavior is provided in Table 2, which reports the electrical parameters as a function of the temperature difference across the TEG. At a minimal ΔT of 0.5 °C, the device generated only 6.26 μW, while a ΔT of 5 °C yielded 4.72 mW. This scaling behavior confirms that even modest improvements in thermal coupling or gradient enhancement can substantially increase the harvested power.

#### Analytical Thermal Circuit and Impedance Matching

To interpret the measured operating point in terms of thermal impedance matching, we consider a lumped thermal circuit of the harvesting stack (heat source → interfaces → TEG → heat sink with forced convection). The steady-state heat flow $\dot{Q}$ can be expressed as

(4) $$\dot{Q}\approx \frac{\Delta T_{\mathrm{env}}}{R_{\mathrm{ext}}+R_{\mathrm{TEG}}},$$

where $\Delta T_{\mathrm{env}}$ denotes the effective temperature driving term between the hot-side environment (motor/adapter vicinity) and ambient air, $R_{\mathrm{TEG}}$ is the effective thermal resistance of the packaged TEG module (including ceramics and thermoelectric legs), and $R_{\mathrm{ext}}$ aggregates the remaining resistances of the external environment,

(5) $$R_{\mathrm{ext}}≜R_{\mathrm{hot}}+R_{\mathrm{int},\mathrm{hot}}+R_{\mathrm{int},\mathrm{cold}}+R_{\mathrm{cold}},$$

with $R_{\mathrm{hot}}$ capturing source-side spreading/conduction effects, $R_{\mathrm{cold}}$ representing the heat-sink-to-air resistance dominated by forced convection in the fan-assisted air tunnel, and $R_{\mathrm{int},\mathrm{hot}},R_{\mathrm{int},\mathrm{cold}}$ accounting for interface/contact contributions.

The measured temperature drop across the module, $\Delta T_{\mathrm{TEG}}$, is related to the heat flow by

(6) $$\Delta T_{\mathrm{TEG}}\approx \dot{Q}\;R_{\mathrm{TEG}}.$$

Combining (4)–(6) yields

(7) $$\frac{\Delta T_{\mathrm{TEG}}}{\Delta T_{\mathrm{env}}}\approx \frac{R_{\mathrm{TEG}}}{R_{\mathrm{ext}}+R_{\mathrm{TEG}}},$$

showing that a non-negligible fraction of the available environmental temperature driving term appears across the TEG when $R_{\mathrm{TEG}}$ is of the same order as $R_{\mathrm{ext}}$. This corresponds to the practical thermal impedance matching regime for maximising extractable power in packaged modules, often summarized by the order condition

(8) $$R_{\mathrm{TEG}}∼R_{\mathrm{ext}}.$$

While the present study does not perform geometry-level optimization of thermoelectric legs (height, fill factor) for the specific convection field (which would require dedicated thermo-mechanical design and/or detailed thermal modeling), the observed stable $\Delta T_{\mathrm{TEG}}$ under fan-assisted convection and the sustained energy-positive operation indicate that the system is not dominated by a single extreme bottleneck resistance. Therefore, the selected packaged TEG and the implemented forced convection heat sink operate in a feasible matching regime for the demonstrated proof-of-concept.

### 4.2. IoT Sensor Analysis

To verify the feasibility of fully autonomous operation, the developed harvester was deployed to power a wireless sensor node integrated into the production line environment. The node consisted of a microcontroller and a LoRa transceiver, executing an energy-aware algorithm designed to regulate data transmissions according to the instantaneous energy state. The experiment aimed to demonstrate whether the harvested energy could reliably sustain continuous operation without external supply.

**Figure 10:**
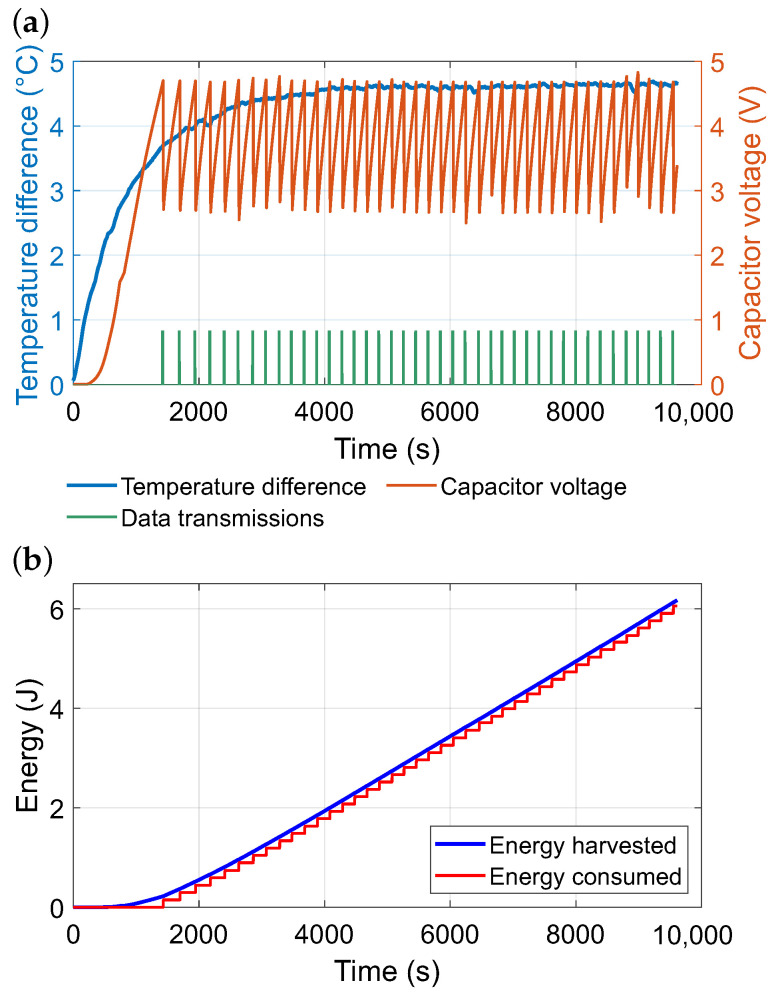
(**a**) Temperature difference and capacitor voltage measured during the experiment with the energy harvester powering a LoRa module and microcontroller. (**b**) Cumulative harvested and consumed energy during the experiment.

Figure 10a shows the temporal evolution of the temperature difference across the thermoelectric module, the corresponding capacitor voltage, and the instants of data transmission. After an initial charging phase, the system entered stable operation, with regular transmissions sustained by harvested energy. The cumulative energy balance presented in Figure 10b confirms that 6.17 J of energy was harvested while 6.05 J was consumed, yielding a slight surplus. This margin indicates that the device was able to compensate for natural fluctuations in the thermal gradient and operate continuously without external intervention.

**Figure 11:**
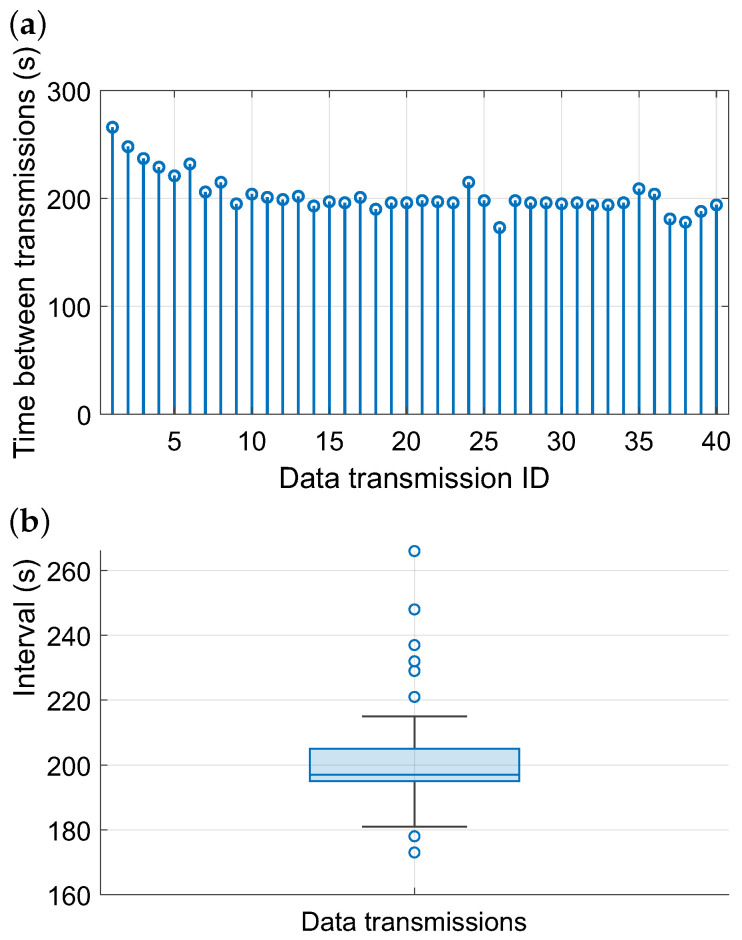
LoRaWAN communication during the factory deployment: (**a**) time series of the storage capacitor voltage $V_{s}$ and the corresponding temperature gradient across the module $\Delta T_{\mathrm{TEG}}$; (**b**) transmission intervals between consecutive uplink messages. Motor load and internal fan speed were not directly instrumented; therefore, $\Delta T_{\mathrm{TEG}}$ is used as a proxy capturing the combined effect of operating and convective conditions. Consistently, higher $\Delta T_{\mathrm{TEG}}$ corresponds to faster energy replenishment and enables shorter transmission intervals, as reflected by the duty-cycled operation.

In Figure 11, the transmission intervals of the wireless sensor node powered by the energy harvester are analyzed. Panel (**a**) illustrates the sequential order of transmissions, where the vertical axis represents the elapsed time between successive messages. Most intervals fall within the range of approximately 170–220 s, with only a few instances deviating towards longer or shorter delays. The box-plot in panel (**b**) provides a statistical summary of the interval distribution. The median lies around 200 s, and the inter-quartile range is relatively narrow, indicating stable sensor operation.

Finally, Table 3 quantifies the dependence of transmission periodicity on the available temperature difference. At ΔT=0.5 °C, the interval between transmissions is estimated to approach 2000 min, whereas at ΔT=5 °C the node is capable of transmitting every 2.6 min. It should be noted that these values were not obtained from direct long-term measurements but were derived analytically: the transmission interval was approximated as inversely proportional to the harvested power, using the experimentally verified operating point at ΔT=4.5 °C as the reference. All other entries in the table, therefore, represent extrapolated estimates based on this proportionality.

Although Figure 11 shows fluctuations in the LoRaWAN transmission intervals, the motor load and the internal fan speed were not instrumented during this proof-of-concept campaign; therefore, a direct one-to-one mapping to these two variables cannot be provided. However, in the presented setup the temperature gradient across the module, $\Delta T_{\mathrm{TEG}}$, is a practical proxy that captures the combined effect of operating conditions (increased waste heat under higher load) and heat rejection conditions (forced convection via the motor fan and the air tunnel). To make this relationship explicit, we additionally report a correlation between the time between transmissions Δt and the average $\Delta T_{\mathrm{TEG}}$ observed in the preceding interval. The resulting trend confirms that shorter transmission intervals occur when $\Delta T_{\mathrm{TEG}}$ is higher, which is consistent with the energy-aware duty-cycling logic: increased available thermal driving leads to a higher harvested power, faster energy replenishment in the storage element, and thus more frequent transmissions. A dedicated campaign including direct logging of motor load and fan speed is identified as future work.

Regarding link-level communication quality, the bit error rate (BER) cannot be directly extracted from the deployed LoRaWAN node because the transceiver does not expose the raw demodulated bitstream after forward error correction; a BER analysis would require specialized instrumentation (e.g., SDR-based reception and controlled channel conditions). Therefore, in this work, we assess wireless reliability using packet-level metrics—successfully received uplink frames that pass integrity checks (CRC) at the receiver. During the presented deployment, all scheduled uplinks were received successfully (i.e., no packet loss was observed in the measurement window), which indicates robust communication under the tested factory conditions. A dedicated BER measurement campaign is identified as future work.

### 4.3. Extended Robustness Experiment

An extended long-run measurement lasting 72.23 h is performed to characterize the temporal evolution of the thermoelectric temperature gradient across the TEG and the long-term behavior of the energy storage stage. The logged variables include hot- and cold-side temperatures, the instantaneous thermal gradient ΔT(t), the TEG terminal voltage $U_{\mathrm{TEG}}\left(t\right)$, the storage-capacitor voltage $V_{C\_\mathrm{STORE}}\left(t\right)$, and the stored energy $E_{C\_\mathrm{STORE}}\left(t\right)=\frac{1}{2}C_{s}V_{C\_\mathrm{STORE}}^{2}$ with $C_{s}=1\;F$. The median logging period is 2.65 s. The log contains an explicit link deactivation event at $t_{\mathrm{off}}$ =71.84 h.

A 1 F storage capacitor was intentionally used in this long-run experiment to stress test the charging capability over extended operation; using the 20 mF capacitor would have resulted in overly rapid charge/discharge cycles, which is not representative for a 72 h characterization and would reduce the interpretability of long-term behavior.

A warm-up time $t_{\mathrm{wu}}$ is defined as the earliest time when the median of a subsequent 10 min window of ΔT(t) falls within ±2% of the long-run median (evaluated over t≥ 1 h and prior to $t_{\mathrm{off}}$). Based on this criterion, $t_{\mathrm{wu}}$ = 54.8 min. Unless stated otherwise, all steady-state statistics are computed over $t\in [t_{\mathrm{wu}},t_{\mathrm{off}}]$.

Table 4 summarizes the steady-state distribution of the thermal gradient. The thermal gradient remains highly stable over the long run (median 4.918 °C; IQR 0.043 °C), indicating limited slow drift within the 72.23 h window.

The storage capacitor behavior is summarized in Table 5. The storage capacitor reaches a peak of 5.161 V (corresponding to 13.319 J), followed by a discharge regime later in the run, which is reflected by the wide spread of $V_{C\_\mathrm{STORE}}$ and $E_{C\_\mathrm{STORE}}$.

Packet-level transmission logs (success/failure) are not available for this 72.23 h dataset; therefore, communication robustness is reported as an estimated feasibility derived from (i) the nominal reporting interval observed in the short experiment and (ii) the measured storage voltage trajectory as a proxy for availability of a valid supply rail. In the short experiment, 41 successful LoRa transmissions are achieved over 9612 s, corresponding to a nominal reporting interval

$$T_{0}=\frac{9612\;s}{41}=234.44\;s\approx 3.91\;\min.$$

Over the steady-state duration of the 72.23 h run, this implies N≈1110 nominal transmission opportunities.

## 5. Discussion

The results of this study demonstrate the feasibility of powering an autonomous IoT device exclusively by harvesting waste heat from an industrial motor. The developed prototype achieved stable operation under real factory conditions, where modest temperature differences of approximately 4–5 °C were sufficient to sustain continuous data acquisition and regular wireless transmissions. Throughout the measurement campaign, the energy harvested exceeded the consumption, enabling uninterrupted functionality of the sensor node. These findings confirm that even small and naturally occurring thermal gradients can be effectively exploited to ensure reliable operation of embedded electronics without the need for batteries or external power sources.

When compared with previously published approaches, the developed prototype demonstrates a favorable balance between harvested energy, power output, and system simplicity. As summarized in Table 6, Santos [14] reported 0.909 J harvested from an electric motor using a single TEG; however, their setup employed a substantially larger thermoelectric module, whereas our prototype operates with a TEG of approximately 60% of that active area. In addition, their wireless transmission relied on the relatively energy-efficient IEEE 802.15.4 protocol, while our system must accommodate a more demanding communication scheme, which further increases the energy budget required for each measurement cycle. Oliveira achieved 370.10 µW with three modules [16] and 320 µW with a hybrid solution [17]. Reeh et al. [15] obtained 1.3 mW by positioning a module between an electric motor and a centrifugal pump, though at a significantly higher motor rating of 2.5 kW. In contrast, the system presented here harvested 6.17 J during 9612 s of operation and delivered an average of 0.64 mW, with peaks exceeding 5 mW, despite being attached to a motor of only 0.25 kW. These results highlight an improved energy harvesting efficiency relative to the scale of the machine and confirm that reliable wireless communication can be sustained even under modest thermal conditions. The findings further underline the benefit of direct integration with the motor housing and utilization of the built-in cooling fan, which enables both stable thermal gradients and minimal installation effort compared with more complex experimental arrangements.

The presented design also introduces several innovations that enhance its practical value for industrial applications. Unlike many studies that rely on multiple modules or large thermal gradients to achieve usable power levels [16,17], this prototype demonstrates that a single TEG can provide sufficient and stable output when integrated directly on the motor casing and supported by the existing airflow of the built-in cooling fan. As indicated in Table 6, the achieved energy and power levels are competitive with or superior to more complex setups, despite the use of a comparatively small motor. A further novelty lies in the use of a hybrid DC/DC conversion chain (LTC3109 and TPS62840), which effectively bridges the ultra-low input voltage of the TEG with the operating requirements of modern microcontrollers and wireless transceivers. Combined with an adaptive transmission strategy, this ensures efficient utilization of the harvested energy and predictable operation of the sensor node. These features make the approach particularly suitable for retrofitting into existing industrial environments, where low cost, non-invasive integration, and long-term autonomy are critical requirements.

Despite these promising results, several limitations of the presented work should be acknowledged. The absolute power output remains in the order of milliwatts, which constrains the range of potential applications to low-power sensing and communication tasks. The experiments were performed on a single motor type under stable operating conditions, and thus the generalisability of the results to different machines or more dynamic environments remains to be validated. In addition, the duration of the experimental campaign was limited to a few hours, whereas long-term stability and reliability over months or years are critical for industrial deployment. Potential issues such as the thermal cycling of the TEG, aging of capacitors, or mechanical stability of the mounting under vibrations were not addressed. In addition, the long-term stability of the thermal interface material (TIM) was not evaluated. Under prolonged vibration and repeated thermal cycling, thermal pastes may exhibit aging (e.g., drying), pump-out, or micro-cracking, which would increase the effective contact thermal resistance. An increase in interface resistance would reduce the effective temperature drop across the TEG and consequently lower harvested power and extend the recharge time between transmissions. In future work, we plan deployment-grade reliability tests combining extended operation, representative vibration exposure, and thermal cycling, and we will compare alternative interface solutions (e.g., controlled clamping force with more robust TIMs such as phase change materials or compliant thermal pads/graphite sheets). These factors represent important aspects for future studies to ensure the robustness of the system in practical use.

Future improvements of the proposed concept may build on both circuit-level and system-level enhancements. At the circuit level, next-generation DC/DC converters such as the EM8900, which can start harvesting from input voltages as low as 5 mV [33], or the MATRIX Mercury with a 9 mV threshold [34], offer opportunities to extend the operational envelope towards smaller temperature gradients and faster cold-start behavior. At the system level, hybrid harvesters that combine thermoelectric conversion with alternative sources such as vibrations, solar radiation, or electromagnetic induction could further increase the available energy budget. Long-term testing across different motor types, varying workloads, and seasonal conditions will be essential to validate durability and scalability. Finally, integration with artificial intelligence techniques for adaptive duty-cycle control and predictive diagnostics represents a promising direction, enabling sensor nodes not only to operate autonomously but also to contribute actively to advanced maintenance strategies in Industry 4.0.

The proposed system uses a commercially available bulk Bi_2_Te_3_ thermoelectric module. Although bulk Bi_2_Te_3_ is commonly associated with applications operating at larger temperature gradients, its selection here is primarily driven by deployment constraints of retrofitting onto industrial machinery: availability in robust packaged modules, reproducible electrical and mechanical interfaces, and practical integration with a curved motor housing and a forced convection heat sink. In other words, the objective of this work is system-level feasibility (energy balance and autonomous operation of a batteryless node) rather than material-level optimization of thermoelectric transport parameters.

It is important to note that, in the low-ΔT regime, the delivered electrical power depends not only on the intrinsic material figure-of-merit but also on the effective module-level quantities (contact resistances, parasitic heat paths, and the achieved ΔT across the ceramic plates). In this context, microstructural factors such as crystal orientation and grain boundary scattering may affect the effective Seebeck coefficient α and the effective thermal conductivity κ, and thus influence the open-circuit voltage ($V_{\mathrm{oc}}\approx \alpha \;\Delta T$) and the internal thermal leakage. For example, an “orientation factor” *F* (with 0<F≤1) can be interpreted as a reduction in the effective Seebeck response due to suboptimal alignment of grains with respect to the heat-flow direction, which would scale the effective Seebeck coefficient as $\alpha_{\mathrm{eff}}\approx F\;\alpha$. Similarly, increased grain boundary scattering can reduce the lattice component of thermal conductivity, potentially improving the effective ΔT across the thermoelectric legs; however, it may also increase electrical resistivity, which can offset gains in α at the module level.

Nanostructured and oriented thin-film materials can therefore offer improved transport performance in principle, particularly when engineered to reduce lattice thermal conductivity while maintaining electrical conductivity. Nevertheless, these approaches introduce additional practical trade-offs for the presented use case: thin-film devices typically provide lower absolute power due to limited active thickness/area, require specialized packaging and low-resistance contacts, and can be less tolerant to mechanical stress and mounting-induced non-uniform pressure. For industrial retrofits where mechanical robustness, repeatability, and cost are key, packaged bulk modules remain a pragmatic choice for demonstrating feasibility. A material-optimized design (including oriented/nanostructured modules and a dedicated characterization of $\alpha_{\mathrm{eff}}$, $\kappa_{\mathrm{eff}}$, and contact resistances under ultra-low ΔT) is identified as future work once the system-level feasibility has been established.

From a practical perspective, the findings highlight the potential of thermoelectric energy harvesting as a sustainable alternative to battery-powered sensing in industrial environments. The elimination of batteries reduces maintenance requirements, lowers operational costs, and minimizes environmental impact associated with large-scale battery disposal. The compact design and straightforward installation make the system attractive for retrofitting onto existing machinery without interrupting operation. By ensuring continuous data collection and transmission, the approach directly supports predictive maintenance strategies, which are central to modern manufacturing concepts. As such, the presented solution not only advances the technical feasibility of battery-free sensing but also contributes to the broader goals of Industry 4.0 by enabling reliable, autonomous, and environmentally responsible monitoring of industrial assets.

A further practical consideration is the form factor and scalability of the mechanical interface. The present prototype uses a rigid aluminum adapter (HTP) primarily to ensure repeatable mounting on a curved motor housing and to maintain controlled contact pressure, while also providing a mechanically stable integration with the heat sink and the fan-assisted air tunnel. This rigid approach has clear trade-offs: it can increase mass and requires a geometry-specific contact surface, meaning that a truly “universal” one-size-fits-all interface across industrial equipment with different diameters and surface curvatures is not realistic without adaptation. However, this is not a fundamental bottleneck for scaling the concept. In practice, scalability is achieved by re-parameterising the interface geometry (e.g., modifying the adapter curvature/radius or using modular adapter inserts) while preserving the same system architecture (TEG + storage + duty-cycled radio).

Compared to rigid adapters, flexible TEG concepts (or compliant interlayers combined with thinner heat spreaders) may better conform to varying curvatures and reduce assembly mass, potentially improving portability. On the other hand, flexible designs introduce their own engineering challenges for this use case: achieving low and stable contact resistance under vibration, ensuring uniform pressure distribution to avoid local air gaps, maintaining mechanical robustness and environmental protection, and integrating an efficient heat sink with forced convection. Therefore, the rigid HTP used here should be viewed as a pragmatic retrofit-oriented solution that enables repeatability and robust mounting for a proof-of-concept. Future work will investigate lighter and more compliant interfaces (e.g., graphite sheets, thermal pads, or hybrid rigid–compliant designs) to broaden applicability across a wider range of curved industrial surfaces while maintaining the effective thermal gradient across the module.

## 6. Conclusions

This work presented the development and validation of a thermoelectric energy harvesting system designed to power wireless sensor devices directly from the waste heat of an industrial electric motor. The prototype demonstrated that even modest temperature gradients of approximately 4–5 °C are sufficient to sustain continuous autonomous operation, including regular wireless data transmissions. Over a measurement period of 9612 s, the system harvested 6.17 J of energy while consuming 6.05 J, maintaining a positive energy balance and confirming the feasibility of battery-free operation under realistic factory conditions.

In comparison with related studies, the proposed approach achieves competitive or superior performance in terms of harvested energy and power output, despite relying on a single thermoelectric module and operating at lower motor power. The integration strategy—direct attachment to the motor housing with the aid of the built-in cooling fan—ensures both stable thermal gradients and simple, low-cost installation, making the solution well suited for retrofitting in industrial environments.

The system further benefits from a hybrid DC/DC power conversion chain, which bridges the ultra-low input voltages of the TEG with the requirements of modern microcontrollers and radio transceivers. Together with an adaptive transmission strategy, this enables predictable energy management and reliable wireless communication.

Looking ahead, the adoption of next-generation converters capable of harvesting from lower input voltages, as well as the combination with hybrid energy sources, offers promising opportunities to expand the operational envelope. Long-term testing across different machine types and workloads will be essential to validate durability. By demonstrating a practical pathway towards sustainable, maintenance-free sensing, this study contributes to the broader objectives of Industry 4.0 and highlights the role of thermoelectric harvesting in enabling reliable and environmentally responsible monitoring of industrial assets.

## Figures and Tables

**Table 1:** Representative studies on TEGs powering wireless sensor nodes, showing main heat sources, number of modules, and reported output.

Study/Experiment	Main Source of ΔT	TEG Used	Harvested Energy	Achieved Power
Elforjani [11]	Gearbox + Air	1	–	–
Ahn [12]	Bearing + Air	1	16.6 J (3400 s)	19.3 mW
Risseh [13]	ATS/EGR	224/240	–	245–420 W; 92–403 W
Santos [14]	Electric motor + Air	1	909.35 mJ	–
Nils [15]	Electric motor + Centrifugal pump	1	–	1.3 mW
Oliveira [16]	Electric motor + Air	3	–	370.10 µW
Kim [18]	PTC heater (car engine)	1	–	7.619 mW
Yu [19]	Switch cabinet/Ambient air	1	–	–
Boitier [20]	Smoke box + Air (coking plant)	1	400 J (one cycle)	800 mW
Toan [21]	Thermos pot + Ambient air	1	–	95.9 mW
Puluckul [22]	Soil + Ambient air	1	–	875 µW (TEG); 337 µW (LTC3109)
Oliveira [17]	Electric motor + Air	1	–	320 µW
Kürschner [23]	Soil/Asphalt pavement + Air/Solar	4	286.24 J/day	20.20 mW
Yan [24]	Human breath	1	–	4.5 µW
Patra [25]	Waste heat + PCM	16	–	502 mW
Lv [26]	Human body heat to Air	1	–	110.2 µW
Boebel [27]	Water pipe + Surrounding soil	2	21 J/day (avg.)	–
Iezzi [28]	Pipe insulation + Air	–	–	0.2 mW
Jiang [29]	General heat	1	–	0.0478 W
Aragones [30]	Steam pipeline + Air	1		1 W/170 °C
Boegel [31]	Coolant + Air	1 –	–	–

**Table 2:** Electrical characteristics of the TEG harvester as a function of the temperature difference Δ*T*: output voltage $V_{\mathrm{TEG}}$, current $I_{\mathrm{TEG}}$, and power $P_{\mathrm{TEG}}$.

Δ*T* (°C)	$V_{\mathrm{TEG}}$ (mV)	$I_{\mathrm{TEG}}$ (mA)	$P_{\mathrm{TEG}}$ (µW)
0.5	2.63	1.75	6.26
1	10.08	6.43	69.25
1.5	11.62	7.30	92.32
2	16.43	10.34	193.82
2.5	31.63	19.96	673.08
3	44.07	27.79	1257.44
3.5	55.73	35.15	1974.95
4	66.54	41.95	2800.47
4.5	76.52	48.23	3696.60
5	86.57	54.54	4724.53

**Table 3:** Estimated time interval between successive transmissions of the IoT node as a function of the temperature difference ΔT.

ΔT (°C)	0.5	1	1.5	2	2.5	3	3.5	4	4.5	5
Estimated time (min)	1976	179	134	64	18	10	6.1	4.4	3.3	2.6

**Table 4:** Steady-state summary statistics of the thermal gradient and TEG voltage during the 72.23 h experiment ($t\in [t_{\mathrm{wu}},t_{\mathrm{off}}]$).

Signal	Mean	SD	Median	IQR	P5	P95
ΔT (°C)	4.918	0.034	4.918	0.043	4.865	4.973
$U_{\mathrm{TEG}}$ (mV)	121.739	3.189	120.184	5.153	118.389	127.015

**Table 5:** Steady-state summary statistics of the storage capacitor voltage and energy during the 72.23 h experiment ($t\in [t_{\mathrm{wu}},t_{\mathrm{off}}]$).

Signal	Mean	SD	Median	IQR	P5	P95
$V_{C\_\mathrm{STORE}}$ (V)	3.732	1.091	3.980	1.754	1.674	5.147
$E_{C\_\mathrm{STORE}}$ (J)	7.559	3.789	7.922	6.624	1.401	13.247

**Table 6:** Comparison of energy harvesting studies which use waste energy from electric motors.

Study/Experiment	Main Source of ΔT	TEG Used	Harvested Energy	Achieved Power
Santos [14]	Electric motor + Air	1	0.909 J	3.19 mW/3.27°
Nils [15]	Electric motor + Centrifugal pump	1	-	1.3 mW
Oliveira [16]	Electric motor + Air	3	-	370.10 µW
Oliveira [17]	Electric motor + Air	1	-	320 µW
This experiment	Electric motor + Air	1	6.1686 J (9612 s)	0.6418 mW (avg.)

## Data Availability

All datasets and source code used in this study are openly available in the Zenodo repository at the following DOI: https://doi.org/10.5281/zenodo.17670801.

## Nomenclature

The following abbreviations are used in this manuscript:

$T_{h}$	Effective hot-side temperature near motor/adapter contact [°C]
$T_{c}$	Effective cold-side temperature near heat sink/air tunnel [°C]
$T_{a}$	Ambient air temperature [°C]
$\Delta T_{\mathrm{TEG}}$	Temperature drop across the TEG module, $\Delta T_{\mathrm{TEG}}=T_{h}-T_{c}$ [°C]
$\dot{Q}$	Heat flow through the harvesting stack [W]
$R_{\mathrm{TEG}}$	Effective thermal resistance of the packaged TEG module [K/W]
$R_{\mathrm{ext}}$	Aggregate external thermal resistance (source + interfaces + heat sink) [K/W]
$R_{\mathrm{par}}$	Aggregate parasitic (shunt) thermal resistance [K/W]
$\eta_{\mathrm{TEG}}$	Fraction of heat flowing through the TEG path (vs. shunt) [–]
$V_{\mathrm{oc}}$	Open-circuit voltage of the TEG [V]
$V_{\mathrm{TEG}}$	TEG terminal voltage under load [V]
$I_{\mathrm{TEG}}$	Current delivered by the TEG under load [A]
$P_{\mathrm{TEG}}$	Electrical power generated by the TEG, $P_{\mathrm{TEG}}=V_{\mathrm{TEG}}I_{\mathrm{TEG}}$ [W]
$V_{s}$	Storage capacitor voltage [V]
$C_{s}$	Storage capacitance [F]
$E_{s}$	Stored energy, $E_{s}=\frac{1}{2}C_{s}V_{s}^{2}$ [J]
$\Delta E_{s}$	Change in stored energy over an interval [J]
$P_{\mathrm{avg}}$	Average available power from energy balance, $P_{\mathrm{avg}}\approx \Delta E_{s}/\Delta t$ [W]
Δt	Time interval (e.g., between transmissions) [s]
